# Repolarization of Tumor-Infiltrating Myeloid Cells for Augmentation of CAR T Cell Therapies

**DOI:** 10.3389/fimmu.2022.816761

**Published:** 2022-02-16

**Authors:** Weichuan Luo, John V. Napoleon, Fenghua Zhang, Yong Gu Lee, Bingbing Wang, Karson S. Putt, Philip S. Low

**Affiliations:** Department of Chemistry, Purdue Institute for Drug Discovery and Purdue Center for Cancer Research, Purdue University, West Lafayette, IN, United States

**Keywords:** CAR T cells, combinational immunotherapy, TLR7 agonist, tumor associated macrophage (TAM), reprograming tumor microenvironment, myeloid-derived suppressor cell (MDSC), tumor microenvironment (TME)

## Abstract

Although CAR T cell therapies have proven to be effective in treating hematopoietic cancers, their abilities to regress solid tumors have been less encouraging. Mechanisms to explain these disparities have focused primarily on differences in cancer cell heterogeneity, barriers to CAR T cell penetration of solid tumors, and immunosuppressive microenvironments. To evaluate the contributions of immunosuppressive tumor-associated macrophages (TAMs) and myeloid-derived suppressor cells (MDSCs) on CAR T cell efficacies, we have exploited the ability of a folate-targeted Toll-like receptor 7 agonist (FA-TLR7-1A) to specifically reactivate TAMs and MDSCs from an immunosuppressive to pro-inflammatory phenotype without altering the properties of other immune cells. We report here that FA-TLR7-1A significantly augments standard CAR T cell therapies of 4T1 solid tumors in immune competent mice. We further show that co-administration of the FA-TLR7-1A with the CAR T cell therapy not only repolarizes TAMs and MDSCs from an M2-like anti-inflammatory to M1-like pro-inflammatory phenotype, but also enhances both CAR T cell and endogenous T cell accumulation in solid tumors while concurrently increasing their states of activation. Because analogous myeloid cells in healthy tissues ar not altered by administration of FA-TLR7-1A, no systemic activation of the immune system nor accompanying weight loss is observed. These data argue that immunosuppressive myeloid cells contribute prominently to the failure of CAR T cells to eradicate solid tumors and suggest that methods to reprogram tumor associated myeloid cells to a more inflammatory phenotype could significantly augment the potencies of CAR T cell therapies.

## Introduction

CAR T cell therapies have revolutionized the treatment of hematopoietic cancers by focusing the cytotoxic potential of a patient’s T cells on his/her cancer cells (1). CAR T cell therapies, however, have been less effective in treating solid tumors due in large part to a tumor microenvironment (TME) that can inhibit the tumoricidal properties of T cells (2). Although CAR T cell inactivation may stem from multiple immunosuppressive stimuli, tumor-associated macrophages (TAMs) and myeloid-derived suppressor cells (MDSCs) are thought to contribute prominently, since they i) secrete immunosuppressive cytokines (e.g. IL-10 and TGF-β) (3, 4), ii) nitrosylate and inactivate T cell receptors (5, 6), iii) express immune checkpoint receptors (7), iv) promote deposition of a dense extracellular matrix that can impede penetration of immune cells (8, 9), and v) produce immunosuppressive enzymes such as arginase 1, CD39 and 5’-nucleotidase (10–12). Based on these activities and data showing an inverse relationship between TAM/MDSC abundances and patient survival (13–15), it is not surprising that efforts to inhibit the immunosuppressive activities of TAMs/MDSCs are increasing (16).

One method for converting TAMs and MDSCs to a more tumor-suppressing (M1-like) phenotype has been to treat the myeloid cells with a TLR7 agonist (17). However, because TLR7 agonists have proven to be too toxic for systemic administration (18–20), they must be targeted specifically to TAMs and MDSCs to avoid unwanted activation of immune cells in healthy tissues. This selective targeting has been recently achieved by linking a TLR7 agonist (TLR7a) to folic acid, that in turn binds to a folate receptor (FRβ) that is almost exclusively expressed on activated myeloid cells (17, 21, 22). Upon endocytosis by TAMs and MDSCs, the folate-TLR7a conjugate (FA-TLR7a) is observed to engage an endosomal Toll-like receptor 7 and initiate signaling pathways that reprogram the TAM/MDSCs into pro-inflammatory M1-like myeloid cells (17, 22).

Based on these considerations, the question naturally arose whether TLR7a-mediated reprogramming of TAMs and MDSCs might create an immune microenvironment that would avoid the usual immunosuppression that plagues CAR T cells in solid tumors. To address this question, we prepared classical anti-CD19 CAR T cells and compared their abilities to eradicate CD19-expressing 4T1 tumors in immune competent mice in the presence and absence of FA-TLR7a. We report here that treatment of the tumor-bearing balb/c mice with FA-TLR7a significantly enhances the infiltration of both CAR T cells and endogenous T cells into 4T1 syngeneic tumors. We further show that the two T cell populations as well as the endogenous TAMs and MDSCs are significantly more activated in the FA-TLR7a treated than nontreated tumors. Based on additional data showing that co-administration of FA-TLR7a significantly augments the abilities of anti-CD19 CAR T cells to eradicate solid tumors *in vivo* without measurable toxicity, we conclude that the targeted reprogramming of TAMs and MDSCs by FA-TLR7-1A can enhance CAR T cell efficacies without activating immune cells in healthy tissues.

## Materials and Methods

### Identification of Syngeneic Murine Tumors That Express Neither a Folate Receptor nor a TLR7 Receptor

To assure that any FA-TLR7a conjugate administered to our tumor-bearing mice could not directly activate any cancer cells, it was important to identify a cancer cell type that expressed neither a folate receptor nor TLR7. For this purpose, several syngeneic cancer cells lines known to grow in immune competent mice were examined for both receptor types. First, 4T1, CT26, and EMT6 cells were cultured in folate-free RPMI 1640 medium (Gibco, Ireland) containing 10% heat-inactivated fetal calf serum and 1% penicillin-streptomycin in 5% CO_2_ at 37°C for one week [since folate-deficient diets can increase expression of folate receptors (23, 24)]. After detaching the cells and incubating them for 1 hour at room temperature in phosphate buffered saline (PBS) containing 10 nM folate-fluorescein, the cells were washed 3x with PBS and analyzed by flow cytometry for folate-fluorescein binding. L1210A cells were treated similarly and used as a folate receptor positive control for these assays.

To test for TLR7 expression, separate flasks of the same 4T1, CT26, and EMT6 cells were detached using 0.25% trypsin and then washed once with PBS. The cells were fixed and permeabilized with Cyto-Fast™ Fix/Perm Buffer Set (BioLegend Cat# 426803) and incubated with anti-mouse CD16/CD32 (BioLegend Cat# 101320) for 15 minutes at room temperature. The cell lines were then incubated with anti-mouse TLR7-PE antibody (BD Biosciences Cat# 565557) for 20 minutes at room temperature prior to washing 2x in 1 ml PBS and analyzing by flow cytometry. 24JK cells were treated similarly and used as a TLR7 positive control for these assays.

### Transduction of Syngeneic Cancer Cell Lines

A lentiviral vector encoding mouse CD19 linked in frame to the green fluorescent protein (GFP) (Sino Biological Cat# MG50510-ACGLN) was assembled in a HEK-293T cell line and employed in combination with polybrene (8µg/mL) to transduce CT26, 4T1, and EMT6 cells. The transduced cells were then sorted by flow cytometry to cells that expressed high levels of both GFP and murine CD19 for further expansion.

When desired, single cell clones were further isolated and proliferated by cloning out single cells from the above CD19^high^ fractions by diluting these fractions to 1 cell/100μl RPMI 1640 media and seeding the single cells into 96 well plates. After growing the cells for ~1 week, the wells with only a single colony were detached with 0.25% trypsin and screened using Opera Phenix™ High Content Screening System to select for clones with high GFP expression. The selected single cell clones were then expanded and stained with anti-mouse CD19-PE antibody (BioLegend Cat# 152407) to identify the single cell clones with the highest CD19-PE fluorescence. The chosen clones were then stored in liquid nitrogen for later studies. Expression levels of CD19 by all three cell lines were periodically confirmed by flow cytometry to assure they retained their high levels of CD19 expression.

### Production of Murine CAR T Cells

Murine anti-CD19 CAR T cells were generated from T cells collected from the spleens of balb/c mice by transducing the T cells with a retroviral vector (MSGV1-1D3-28Z.1-3 mut) kindly provided by James Kochenderfer & Steven Rosenberg (Addgene plasmid # 107227; http://n2t.net/addgene:107227; RRID : Addgene_107227) (25). Splenic T cells were then isolated by negative selection (EasySep™ Cat# 19851) and activated with anti-CD3/CD28-conjugated Dynabeads (Gibco™ Cat# 11453D) for 24 hours prior to their transfer to RetroNectin-coated plates for transduction with the above vector. To boost transfection efficiency, second transduction was performed one day after the first, and the cells were cultured for an additional 3 days. Transduction efficiency was quantitated using an anti-rat IgG F(ab’)_2_ fragment conjugated to Alexa Fluor 594 (Jackson ImmunoResearch Laboratories Cat# 212-586-168).

### Analysis of *In Vitro* Cytotoxicity of Anti-Mouse CD19 CAR T Cells

Evaluation of the ability of anti-mouse CD19 CAR T cells to kill CD19^+^ cancer cells *in vitro* was performed by co-culturing 4T1-mCD19 cancer cells with the above anti-CD19 CAR T cells for 16 hours at 1:1, 2:1, and 5:1 effector to cancer cell ratios. After gently washing the culture wells with PBS to remove unattached cells, 0.25% trypsin was added to each well, and the incubation was continued for 20 min at 37°C before collecting the cells from each well, washing them twice with RPMI 1640 media containing 10% FBS, and incubating them for 15 min with 7-aminoactinomycin D (7-AAD) to distinguish live from dead cells. Cell suspensions from each well were then analyzed by flow cytometry for 7-AAD staining, size, and GFP expression (4T1 cancer cells) to distinguish live from dead cancer cells and CAR T cells.

### Evaluation of the Impact of FA-TLR7-1A on the Efficacy of Ant-CD19 CAR T Cell Therapies in Treating Solid 4T1 Tumors in balb/c Mice

FA-TLR7-1A was prepared by conjugating folic acid to the TLR7 agonist *via* a noncleavable linker using chemistry related to that described elsewhere (22, 26). Six- to eight-week-old female balb/c mice were purchased from Envigo, housed and treated under the Purdue University Animal Care and Use Committee-approved protocols. 4T1-mCD19 cells (5×10^4^) were injected subcutaneously into balb/c mice and allowed to grow to 50 mm^3^ size and randomized into groups prior to initiation of therapy. To prepare a niche for implantation of CAR T cells, the mice were then lymphodepleted by exposure to 4 Gy total body irradiation on Day 0. Treatment with FA-TLR7-1A was also initiated on Day 0 and continued 5 times/week via tail vein injection unless indicated otherwise (the dosing frequency was optimized based on data shown in **Figure S1**, panel **A, B**). Approximately 1 million anti-CD19 CAR T cells were infused intravenously through the lateral tail vein on Day 1, and each 4T1 tumor’s growth was determined every other day using the formula, tumor volume= length x width^2^/2. Cohorts included mice treated with PBS, CAR T cells only, or mice treated with both CAR T cells and FA-TLR7-1A (**Figure S1**, panel **C**). To determine whether any of the treatments might have caused significant systemic toxicity, the weights of all mice were recorded every other day for the duration of the study. For evaluation of the effect of tumor size on the ability of FA-TLR7-1A to enhance anti-CD19 CAR T cell therapy, single cell clones of 4T1-mCD19 cells (5×10^4^) were injected subcutaneously into balb/c mice and allowed to grow to 50, 90, or 130 mm^3^ tumors prior to initiation of therapy as described above.

### Evaluation of the Impact of FA-TLR7-1A Mediated Reprogramming of Tumor Myeloid Cells on the Properties of Tumor-Infiltrating Immune Cells

Upon completion of the above experiments, tumor-bearing mice were euthanized, and associated tumors were digested to afford an unsorted tumor cell suspension. Suspension cells were initially stained with Zombie Violet (BioLegend Cat# 423114) to quantitate cell survival, and after washing, incubated with anti-mouse CD16/CD32 for 20 minutes on ice to block unoccupied Fc receptors. For quantitation of tumor-infiltrating leukocytes, cells were then stained with anti-mouse CD45-FITC antibody (BioLegend Cat# 103108). For quantitation of macrophages and MDSCs, cells were stained with anti-mouse F4/80-APC (BioLegend Cat# 123116), anti-mouse CD11b-Alexa Fluor 700 (BioLegend Cat# 101222), and anti-mouse Gr-1-Alexa Fluor 594 (BioLegend Cat# 108448) antibodies. For the assessment of the iNOS^+^ and Arginase 1^+^ population and their iNOS^+^/Arg 1^+^ ratios, suspension cells were fixed and permeabilized with Cyto-Fast™ Fix/Perm Buffer Set (BioLegend Cat# 426803) prior to staining with anti-mouse arginase-1-PE antibody (eBioscience Cat# 12-3697-82) and anti-mouse iNOS-APC-eFluor 780 antibody (eBioscience Cat# 47-5920-80). To determine the percentages of MDSCs inside the tumors, digested cells were stained with anti-mouse CD11b-APC-eFluor 780 (eBioscience Cat# 47011282) and anti-Gr-1-Alexa-594 (BioLegend Cat# 108448). For quantitation of tumor-infiltrating T cells and CAR T cells, cell suspensions were stained with anti-mouse CD3-APC (eBioscience Cat# 17-0031-83) or anti-rat IgG F(ab’)_2_-Alexa Fluor 594. Finally, to analyze the number of activated T cells and CAR T cells inside the tumors, anti-mouse CD25- PerCP/Cyanine5.5 (BioLegend Cat# 101911) or anti-mouse CD69-PE (BioLegend Cat# 104507) were similarly used. All samples were washed 2x with PBS prior to analysis by flow cytometry.

### Evaluation of the Fraction of Monocytes that Express a Functional FRβ^+^ in Fresh Human Blood

To quantitate the percentages of monocytes that express either a functional or nonfunctional FRβ^+^ in fresh human blood, fresh PBMCs were isolated from five healthy donors following informed consent using Ficoll density gradient centrifugation. After removing erythrocytes with RBC lysis buffer, the PBMCs were stained at 4°C for 1 hour with anti-human PE-CD14 (BioLegend Cat# 301805), fluorescein-labeled anti-human FRβ^+^ (FITC-m909, 10µg/ml), and folate-fluorescein conjugate (EC17, 100nM) in the presence or absence of 100X folate-glucosamine (competitor). All samples were then washed 3x with PBS and analyzed by flow cytometry.

### Flow Cytometry Analysis

Flow cytometry was performed using an Attune™ NxT Acoustic Focusing Cytometer (Invitrogen) and assessed with Attune™ NxT and FlowJo Software.

### Immunohistochemistry of Tumor Sections

Formalin-fixed, 4T1-mCD19 tumors were embedded in paraffin, sectioned, and stained with anti-mouse CD3 antibody in a blinded manner by a histology technologist. Images were obtained using a Leica Versa 8 whole-slide scanner.

### Ethics Approval

All animal care and use were followed by NIH guidelines, and all experimental protocols were approved by the Purdue Animal Care and Use Committee.

### Statistical Analyses

GraphPad Prism version 8 software (Graphpad; San Diego, CA) was used for statistical analyses. All figures report mean ± s.e.m values unless otherwise noted. For comparison of two groups, a 2-tailed t-test was utilized while for comparison of multiple groups a 1-way ANOVA followed by a Tukey *post hoc* analysis was employed where appropriate. (*P < 0.05, **P < 0.01, ***P < 0.001, ****P < 0.0001).

## Results

### Selection of a Tumor Model in Immune Competent Mice

**Figure 1:**
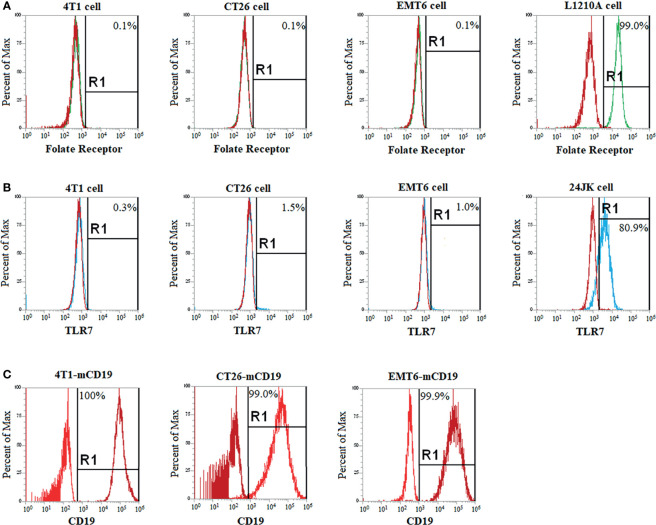
Selection of a CD19^+^ cancer cell line that expresses no folate nor TLR7 receptor and will grow in immune competent mice. Cancer cells lines (4T1, CT26, EMT6) that were known to grow in immune competent mice were cultured for 1 week in folate deficient medium to upregulate inducible folate receptors (if any) and then screened by flow cytometry to assure that the cells still expressed neither a folate receptor **(A)** nor a Toll-like receptor 7 **(B)**. Known folate receptor positive (L1210A cells) and TLR7 positive (24JK cells) were used as positive controls. FR and TLR7 negative cell lines were then stably transduced with murine CD19 (mCD19) to create a cancer cell line that would respond to an anti-CD19 CAR T cell therapy. Successful expression of CD19 was confirmed after one-month of culturing by flow cytometry **(C)**. Each flow cytometry analysis was performed at least three times, with representative histograms shown above.

For evaluation of the impact of TLR7 agonist-mediated repolarization of TAMs and MDSCs on the efficacy of CAR T cell therapies in solid tumors, we required a cancer cell line that would be devoid of both folate receptors and TLR7, so that our FA-TLR7-1A conjugate could neither bind nor directly stimulate the implanted cancer cells. To identify such a cancer cell line, we first screened several syngeneic tumor cell lines for folate receptor expression (FR). As seen in the flow histograms of **Figure 1A**, CT26, 4T1, and EMT6 cells all failed to bind a folate-fluorescein conjugate (compare with folate receptor positive control, L1210A cells), even after culturing in folate deficient medium to try to induce FR upregulation (24, 27), i.e. demonstrating that these cell lines do not express a functional folate receptor (28, 29). Next, to determine whether any of the same cell lines express a Toll-like receptor 7, we fixed, permeabilized, and stained the cells with an antibody to murine TLR7. As seen in the flow cytometry histograms of panel B (**Figure 1B**), none of the cells were observed to express TLR7 (compare with positive control in 24JK cells). Based on these data, we conclude that neither free TLR7-1A nor the FA-TLR7-1A conjugate should be capable of directly activating any of the above cancer cell lines.

Next, to create a tumor model that would be relevant to the commonly used anti-CD19 CAR T cell therapies today, we transduced each of the above cell lines with murine CD19 (mCD19). As described in Methods, lentiviral vector transduction of the cells with mCD19 followed by sorting for high mCD19 expressors (**Figure 1C**) yielded the desired CD19^+^ cancer cell lines. Then, to identify the cancer cell lines with the strongest tumor-forming potential *in vivo*, each of the transduced cell lines was implanted subcutaneously in balb/c mice and allowed to proliferate. Although all cell lines formed stable murine CD19 (mCD19) positive tumors, because 4T1 cells produced the most aggressive tumors with the highest percentage of FRβ^+^ myeloid cells (17, 30), we elected to conduct all further studies using the murine CD19-expressing 4T1 cell clone, hereafter termed 4T1-mCD19.

### Analysis of the Ability of Anti-CD19 CAR T Cells to Kill 4T1-mCD19 Cells *In Vitro* and *In Vivo*

**Figure 2:**
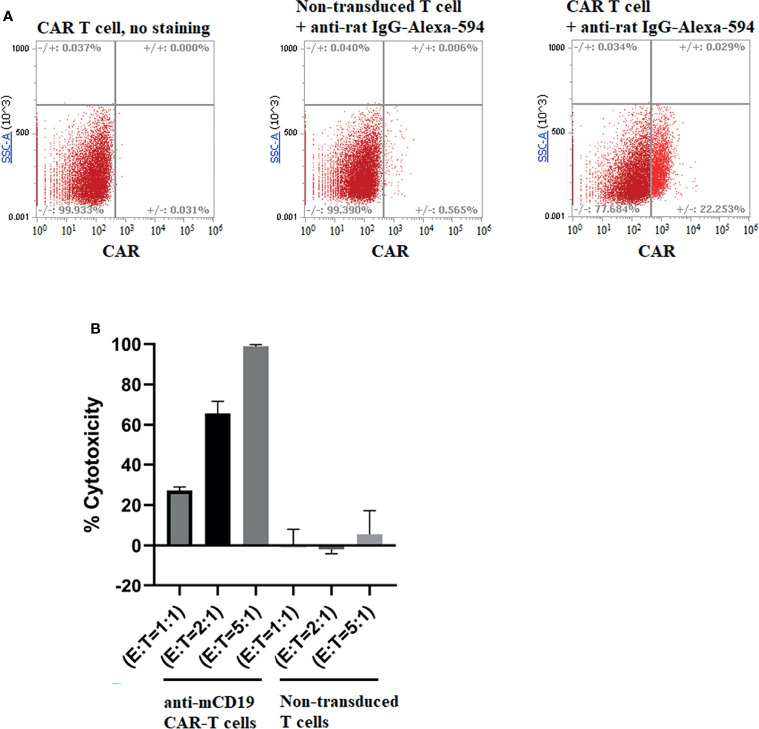
Production of murine CAR T cells that recognize murine CD19 and analysis of their ability to kill 4T1-mCD19 cells *in vitro.* **(A)** Spleen derived T cells were transduced to express an anti-CD19 CAR and then examined for transduction efficiency by flow cytometry using an anti-rat IgG labeled with AlexaFluor 594, as described in Methods. Data shown are representative of three separate transduction procedures. **(B)** Analysis of the ability of the aforementioned anti-mouse CD19 CAR T cells to kill 4T1-mCD19 cancer cells *in vitro*. (Effector:target cell ratio = 5:1). Bar graphs display mean ± S.D. n=3.

To determine whether anti-CD19 CAR T cells could kill 4T1-mCD19 cells *in vitro*, we generated anti-CD19 CAR T cells by transducing murine T cells collected from the spleens of balb/c mice (i.e. the mouse strain from which 4T1 cells are derived) with a retroviral vector containing the anti-CD19 CAR (see *Materials and Methods*). As shown in **Figure 2A**, these anti-CD19 CAR T cells were routinely generated with ~20% efficiency. Moreover, their abilities to kill 4T1-mCD19 cells reached 100% efficiency at an effector to tumor cell ratio of 5:1 (**Figure 2B**), even though nontransduced T cells lacking the anti-CD19 CAR displayed insignificant cytotoxicity against the same CD19^+^ tumor cell clones (panel B).

**Figure 3:**
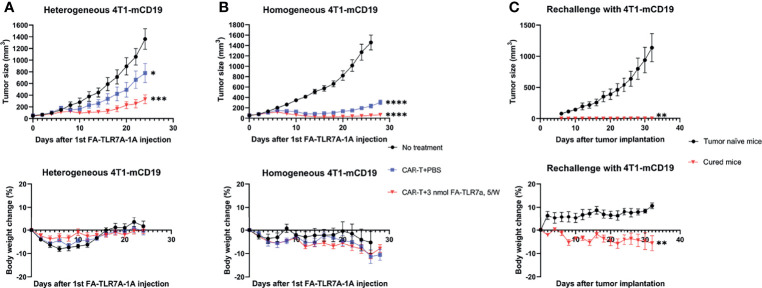
Analysis of the ability of anti-CD19 CAR T cells to kill 4T1-mCD19 cells *in vivo.* **(A)** A “heterogeneous” preparation (not a single cell clone) of 4T1-mCD19” tumor cells was implanted into balb/c mice and allowed to form 50 mm^3^ tumors prior to initiation of therapy either with saline (black line), anti-mCD19 CAR T cells alone (blue line), or anti-mCD19 CAR T cells plus FA-TLR7-1A (red line). Tumor size (upper panel) and total body weight (lower panel) were then monitored as a function of time. **(B)** The studies described in part A were repeated following isolation and expansion of a single cell clone of 4T1-mCD19 cells. **(C)** Mice from panel B that showed no sign of any residual cancer for 30 days after termination of the therapy were rechallenged with fresh 4T1-mCD19 cells and the growth of the resulting tumors was monitored (red line). Tumor naïve mice were similarly injected with the same number of 4T1-mCD19 cells and used as a control (black line). The above studies were conducted at least twice using cohorts of 5 to 9 mice per group each time. More specifically, for the above studies involving heterogeneous 4T1-mCD19 tumors, the data derive from cohorts of 5 mice/group. For the above studies involving homogeneous 4T1-mCD19 tumors, the “No treatment”, “CAR T+PBS”, and “CAR T+3n mol FA-TLR7-1A, 5W” treatment groups contained 5, 6 and 9 mice/group, respectively. For rechallenge with 4T1-mCD19, n=4 for all the groups. Mean ± SEM. Statistical significance between multiple groups was determined using a 1-way ANOVA followed by a Tukey *post hoc* analysis. Statistical significance between two groups was determined using an unpaired two‐tailed t‐test (*P < 0.05, **P < 0.01, ***P < 0.001, ****P < 0.0001).

To evaluate the ability of anti-CD19 CAR T cells to kill CD19^+^ tumor cells *in vivo*, we next implanted balb/c mice with the same preparation of 4T1-mCD19 breast cancer cells and allowed the tumors to reach ~50 mm^3^ in size before infusing the mice intravenously with either saline or anti-CD19 CAR T cells. After segregating the CAR T cell-infused mice into two identical cohorts, we treated one cohort 5 days/week with a folic acid-targeted TLR7 agonist (FA-TLR7-1A) and the other cohort with saline. This folate receptor-targeted TLR7 agonist was chosen for reprogramming of the tumor-associated macrophages (TAMs) and myeloid-derived suppressor cells (MDSCs) because a related FA-TLR7 agonist conjugate had been previously shown to convert M2-like immunosuppressive myeloid cells into M1-like inflammatory myeloid cells in solid tumors (17) without directly affecting any other immune cells in the body. As shown in **Figure 3A** (upper panel), although treatment of the tumor-bearing mice solely with murine anti-CD19 CAR T cells suppressed tumor growth, cotreatment with the same CAR T cells plus FA-TLR7-1A improved this therapeutic potency. Moreover, because this co-administration of FA-TLR7-1A caused no further weight loss, the augmented potency was likely achieved without significant added toxicity.

**Figure 4:**
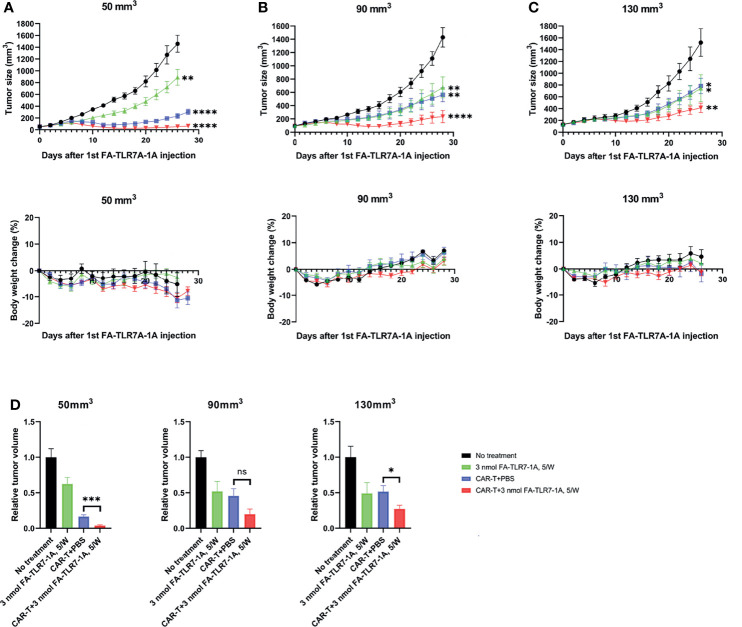
The effect of tumor size on the ability of FA-TLR7-1A to enhance anti-CD19 CAR T cell therapy of solid 4T1-mCD19 tumors. Analysis of tumor size and body weight change of mice in which treatments started when the average tumor sizes reached **(A)** 50 mm^3^, **(B)** 90 mm^3^, and **(C)** 130 mm^3^. **(D)** Comparison of the relative tumor sizes on day 26 after initiation of therapy. The number of mice per treatment group were as follows: for mice with 50mm^3^ tumors: “No treatment”, n=5; “3 nmol FA-TLR7-1A, 5W”, n=6; “CAR T+PBS”, n=6; “CAR T+3n mol FA-TLR7-1A, 5W”, n=9. For mice with either 90mm^3^ or 130mm^3^, n=5 for all the groups. Mean ± SEM. Statistical significance between multiple groups was determined using a 1-way ANOVA followed by a Tukey *post hoc* analysis. Statistical significance between two groups was determined using an unpaired two‐tailed t‐test (*P < 0.05, **P < 0.01, ***P < 0.001, ****P < 0.0001, ns, not significant P ≥ 0.05).

An important TME variable not yet examined in the above studies was tumor size. Thus, research by other laboratories has demonstrated that the immunosuppressive properties of solid tumors change with tumor size, where infiltrating TAMs and MDSCs become both more abundant and more suppressive as tumor size enlarges (31–34). To determine whether the ability of FA-TLR7-1A to augment CAR T cell therapies might also change with tumor size, therapeutic responses were compared among tumors in which initiation of therapy was delayed until their sizes had reached 50 mm^3^, 90 mm^3^, or 130 mm^3^. As shown in **Figure 4**, the contribution of FA-TLR7-1A to inhibition of tumor growth was similar if not greater as tumor size increased; however, the impact of CAR T cell therapy on tumor expansion declined as the tumors enlarged. These data suggest that reprogramming of myeloid cells in the TME may become more important as tumor burden increases. The data may also suggest that CAR T cell therapies, in general, can be improved if solid tumors were maximally debulked before initiation of therapy (35, 36).

### Evaluation of the Mechanism Underpinning Augmentation of CAR T Cell Efficacy With FA-TLR7-1A

Analysis of the residual tumor masses in the above study revealed that the surviving cancer cells were all CD19 negative, suggesting that the absence of completes responses was largely due to loss or downregulation of the mCD19 “tumor antigen” (**Figure S2**). In an effort to prevent such selection for antigen negative cells, we cloned out single 4T1-mCD19 cells and implanted a clone that exhibited high mCD19 expression into balb/c mice to enable their development of homogeneous tumors. As shown in **Figure 3B**, infusion of these mice with the same anti-CD19 CAR T cells promoted a somewhat improved suppression of tumor growth, but again yielded no complete responses. In contrast, co-administration of FA-TLR7-1A to an identical cohort of mice yielded 4 out of 9 complete responses, with all responders remaining tumor-free for at least the 30 subsequent days of monitoring. In fact, rechallenge of the complete responders at the end of the 30-day monitoring period with additional 4T1-mCD19 cells still yielded no detectable tumors despite the absence of any further therapy (**Figure 3C**). These data suggest that the complete responders had developed a sustained immunity to the 4T1-mCD19 cancer cells and that this immune memory required co-administration of FA-TLR7-1A.

**Figure 5:**
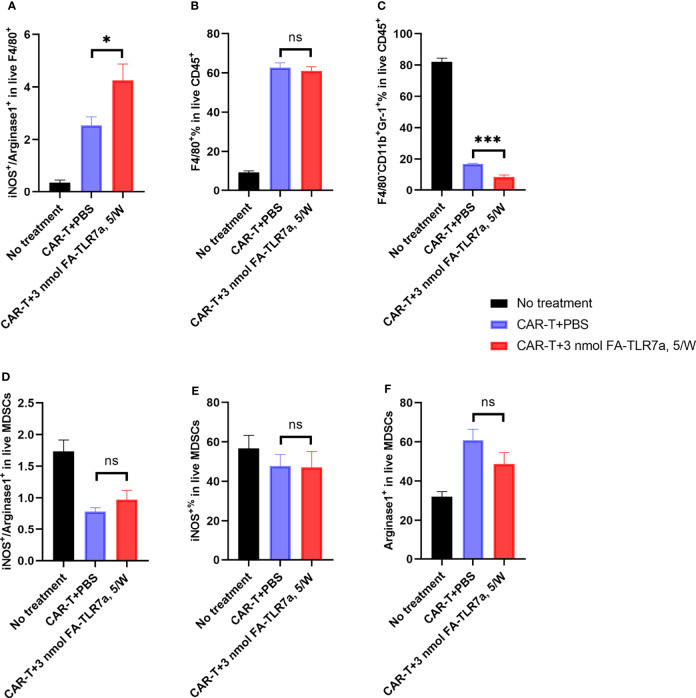
Impact of CAR T cells and FA-TLR7-1A on the properties of tumor infiltrating TAMs and MDSCs. **(A)** Estimation of the M1:M2 ratios of F4/80 positive macrophages within the solid 4T1-mCD19 tumors following treatment with the indicated therapies. The ratio of inducible nitric oxide synthase (iNOS) to arginase 1 (Arg 1) was used as an estimate of M1-like to M2-like macrophages. **(B)** Percentage of live CD45^+^ cells in the solid tumors that were F4/80 positive macrophages. **(C)** Percentage of MDSCs in live CD45^+^ cells in the solid tumors. **(D)** Ratio of the iNOS^+^ to Arg 1^+^ population among live MDSCs. **(E)** Percentage of the iNOS^+^ population in live MDSCs. **(F)** Percentage of Arg 1^+^ population in live MDSCs. This study was performed three times with similar results each time. The number of mice/group is the same as reported in the legend to **Figure 3**, except in the CAR-T + 3 nmol FA-TLR7-1A, 5/W group only 5 of 9 tumors could be analyzed because 4 of 9 mice experienced complete cures. Mean ± SEM. Statistical significance was determined using an unpaired two‐tailed t‐test (*P < 0.05, ***P < 0.001, ns, not significant P ≥ 0.05).

Since the hypothesis underpinning this study was that TLR7-1A-mediated stimulation of TAMs and MDSCs might enhance CAR T cell efficacy by shifting the TME towards a more inflammatory state, we next examined whether the immune cells in the TME might have acquired a more inflammatory phenotype upon FA-TLR7-1A administration. To test this hypothesis, tumor tissue resected upon the termination of the above therapies was subjected to gentle digestion to dissociate cells and analyzed by flow cytometry for immune cell markers (**Figure S3**). As shown in **Figure 5A**, treatment with FA-TLR7-1A nearly doubled the ratio of M1:M2 macrophages (as measured by the iNOS:arginase 1 ratio) and halved the percentage of MDSCs in the live CD45^+^ tumor cell population (**Figure 5C**). There was, however, no significant difference in the percentage of tumor-associated macrophages (**Figure 5B**) or the various subpopulations of live MDSCs (**Figures 5B–F**). Because TAMs and MDSCs constitute the only folate receptor-expressing cells in these solid tumors (17, 37), the results suggest that folate receptor-mediated uptake of FA-TLR7-1A can shift the properties of TAMs and MDSCs to a more tumoricidal phenotype.

**Figure 6:**
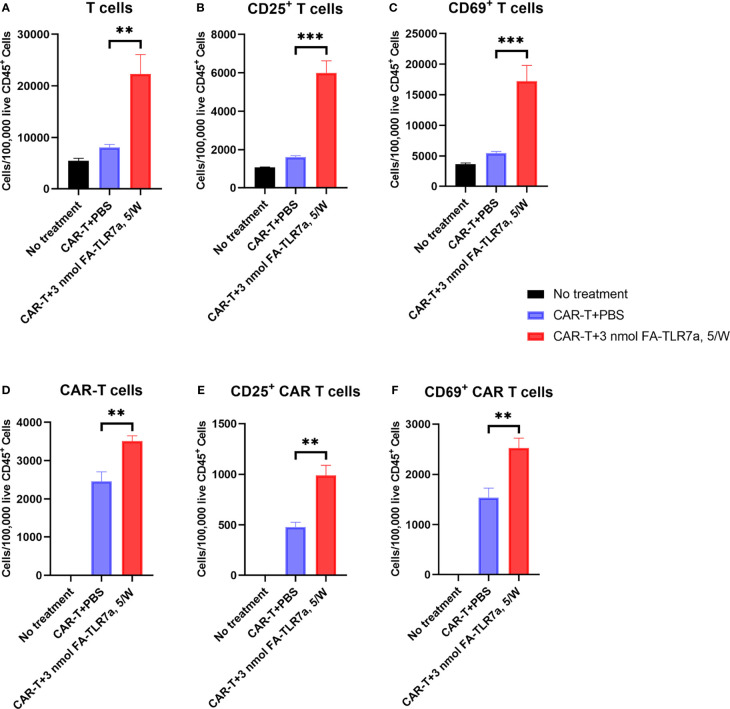
Impact of CAR T cell therapy in the presence and absence of FA-TLR7-1A on the number and activation states of tumor-infiltrating T cells and CAR T cells. **(A–C)** The number of total T cells, CD25^+^ T cells, and CD69^+^ T cells per 100,000 live CD45^+^ cells in the tumors following the indicated treatments. **(D–F)** The number of total anti-CD19 CAR T cells, CD25^+^ anti-CD19 CAR T cells, and CD69^+^ anti-CD19 CAR T cells per 100,000 live CD45^+^ cells in the tumors following the indicated treatments. This study was performed three times with similar results each time. The number of mice/group is the same as reported in the legend to **Figure 3**, except in the CAR-T + 3 nmol FA-TLR7-1A, 5/W group only 5 of 9 tumors could be analyzed because 4 of 9 mice experienced complete cures. Mean ± SEM. Statistical significance was determined using an unpaired two‐tailed t‐test (**P < 0.01, ***P < 0.001).

Next, to investigate whether treatment with FA-TLR7-1A might have also changed the properties of endogenous T cells and/or CAR T cells in the same tumor masses, we also examined the surface markers of these cells by flow cytometry (**Figure S4**). As shown in panels A of **Figure 6**, co-administration with FA-TLR7-1A more than doubled the total number of murine T cells accumulating in the tumors, and many of these T cells, including CAR-T cells, became better effector cells, as evidenced by their enhanced expression of activation markers, CD25 and CD69 (**Figure 6**, panel B-F). Improved T cell infiltration was also observed in FA-TLR7-1A treated tumors using immunohistochemistry (**Figure S5**). Because FA-TLR7-1A is only capable of entering folate receptor-expressing cells (i.e. TAMs and MDSCs), this concomitant activation of both unmodified and genetically engineered T cells suggests that repolarization of the MDSCs and TAMs can promote their release of chemokines and cytokines (38, 39) that in turn can induce a more activated phenotype in the tumor-infiltrating T cells and CAR T cells.

### Assessment of the Specificity of FA-TLR7-1A for TAMs and MDSCs

As noted above, nontargeted TLR7 agonists have proven to be too toxic for systemic administration because they activate most immune cells in the body (18–20). Since none of the FA-TLR7-1A-treated animals in **Figures 3**, **4** showed any weight loss beyond that caused by the CAR T cell therapy, the question next arose whether FA-TLR7-1A might have avoided systemic activation of the immune cells in healthy tissues. To address this question, we collected myeloid cells from the spleens of the same tumor-bearing mice used in **Figure 4** and examined their activation markers by flow cytometry. As shown in **Figure S6**, systemic treatment with FA-TLR7-1A exerted no effect on either the percentage of macrophages in the spleen or their M1/M2 ratios. These results confirm that FA-TLR7-1A is specific for FR-expressing myeloid cells in the tumor microenvironment and that systemic administration of FA-TLR7-1A should have little or no effect on macrophages in healthy tissues.

### Evaluation of FRβ Expression on Peripheral Blood Monocytes From Healthy Human Donors

**Figure 7:**
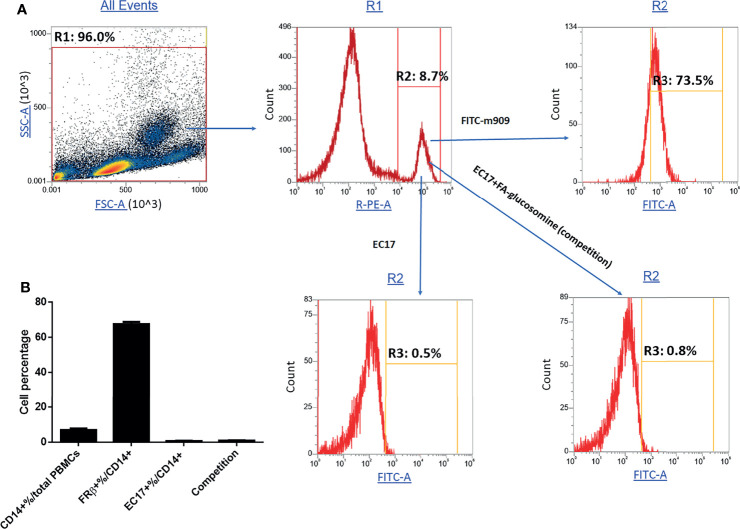
Evaluation of functional and nonfunctional FRβ expression in monocytes from fresh human PBMCs. Fresh PBMCs isolated from healthy donors were stained with anti-human PE-CD14, fluorescein-labeled antibody to human FRβ or folate-fluorescein conjugate (EC17) in the absence or presence of 100X folate-glucosamine (competition) and then subjected to FACS analysis. **(A)** Representative gating strategy for isolating monocytes (CD14^+^%/total PBMCs), total FRβ+ monocytes (FRβ+%/CD14+) and functional FRβ (EC17^+^%/CD14+). **(B)** Percentage of monocytes and their functional and nonfunctional FRβ expression (n=5 human donors).

Because expression of a functional folate receptor is required for binding and internalization of FA-TLR7-1A, it was important to determine whether healthy human donors might express any circulating blood cells with a functional folate receptor (**Figure 7**). Although 73% of circulating CD14^+^ monocytes were found to stain positive for human folate receptor beta (determined by m909 staining), almost none of the cells were found to bind folate-linked fluorescein (EC17), suggesting the folate receptors were nonfunctional. Importantly, these data support previous speculations that a nonfunctional folate receptor might be present on some human myeloid cells but remain nonfunctional until activated by a proinflammatory or anti-inflammatory stimulus (28). This requirement for activation is obviously critical, since the myeloid cells in a solid tumor mass are activated whereas those in healthy tissues are not, i.e. allowing FA-TLR7-1A to only activate the TAMs and MDSCs in the tumor.

## Discussion

TAMs and MDSCs are thought to be major orchestrators of the immunological properties of solid tumors because they i) secrete immunosuppressive cytokines such as TGF-β and IL-10 (3), ii) express checkpoint receptors (e.g. PD-L1) that inhibit effector functions (40), iii) release growth factor such as VEGF, PDGF, and FGF that promote cancer cell and neovasculature proliferation (41, 42), and iv) discharge chemokines (e.g. CCL18) and cytokines (e.g. TGFβ) that recruit fibroblasts and enhance tumor proliferation (4, 43). Not surprisingly, the abundance of TAMs/MDSCs generally correlates with poor patient survival (13–15), leading many to hypothesize that eliminating or reprogramming TAMs and MDSCs should augment immunotherapies (16, 37, 39).

We have undertaken to test this hypothesis by reprogramming tumor-infiltrating myeloid cells to a more inflammatory M1-like phenotype and examining the impact of such reprogramming on the ability of CAR T cells to eradicate solid tumors. We chose FA-TLR7-1A for repolarizing the TAMs and MDSCs because only activated TAMs and MDSCs express functional FRβ, i.e., allowing the membrane impermeable FA-TLR7-1A conjugate to be internalized primarily by these FRβ^+^ tumor-infiltrating cells (17). Thus, the only major cell types besides activated macrophages to express a functional folate receptor in healthy adults are the proximal tubule cells of the kidney (44) and epithelial cells of the choroid plexus (45). Since these two cell types lack TLR7, the only cell lineage with the capacity to both internalize and respond to FA-TLR7-1A should be the FRβ^+^TLR7^+^ myeloid cells. Not surprisingly, the absence of these cells from healthy tissues (17, 46, 47) assures that unwanted systemic activation of the immune system and the accompanying toxicity will be minimal. Moreover, the fact that only FRβ-expressing TAMs/MDSCs are immunosuppressive (17) assures that repolarization of this specific cell population should prove most effective in augmenting a co-administered cancer immunotherapy. Recently, Alba Rodriguez-Garcia et al. reported that anti-FRβ CAR T cells could promote enhanced inhibition of tumor growth by eliminating FRβ expressing TAMs and MDSCs (37). However, the fact that such anti-FRβ CAR T cells should also recognize and destroy most of the circulating monocytes raises a concern that anti-FRβ CAR T cells might cause unwanted systemic immunosuppression. The inability of FA-TLR7-1A to bind nonfunctional FRβ on healthy peripheral blood monocytes while efficiently targeting functional FRβ on TAMs and MDSCs obviously avoids this toxicity.

It was interesting to note that the FA-TLR7-1A-stimulated reprogramming of TAMs/MDSCs also changed the properties of tumor-infiltrating T cells and CAR T cells. Not only did more T cells infiltrate the affected tumors, but the phenotypes of the T cells also became more activated. Because FA-TLR7-1A stimulation is limited to FRβ-expressing tumor myeloid cells, these data argue that repolarization of TAMs/MDSCs to an inflammatory phenotype leads to increased recruitment and activation of infiltrating lymphocytes. A possible decrease in TAM/MDSC expression of checkpoint receptors (e.g. PD-L1) and/or immunosuppressive cytokines (e.g. IL-10, TGFβ) or an increase in expression of pro-inflammatory cytokines (e.g. TNFα, IFNγ) could conceivably account for these changes.

Consistent with observations of others (48), our CAR T cell therapy became less effective as tumor size increased, with the CAR T cell monotherapy reducing tumor growth by approximately 85%, 55%, and 50% in tumors of ~50, 90, and 130 mm^3^ sizes, respectively (**Figure 4**). Surprisingly, treatment with FA-TLR7-1A alone appeared to show the opposite trend, with the targeted conjugate suppressing tumor grow by only ~40% in the 50 mm^3^ tumor cohort, but improving to ~50% in both the 90 and 130 mm^3^ tumor cohorts. While part of this size correlation may derive from an increased probability of encountering a cancer cell with a resistance mutation as tumor size increases, a second component may stem from the fact that the TME becomes increasingly immunosuppressive as tumors enlarge (31–34). Thus, as tumor size increases, the percentage of infiltrating T cells declines (49), the proportion of tumor-promoting cancer-associated fibroblasts increases (50), the polarization of macrophages shifts towards a more anti-inflammatory state (31), and the number of suppressive myeloid cells also rises (34). Although these correlations clearly portend a more refractory tumor as its size increases, they also suggest that myeloid cell repolarization could make an increasingly greater contribution to tumor immunotherapies as malignant lesions enlarge. The fact that most cancers are first diagnosed when tumors already exceed 130 mm^3^ suggests that TME reprogramming may become a critical element in regressing solid tumors in the future.

## Data Availability Statement

The original contributions presented in the study are included in the article/**Supplementary Material**. Further inquiries can be directed to the corresponding author.

## Ethics Statement

The animal study was reviewed and approved by Purdue Animal Care and Use Committee.

## Author Contributions

WL contributed to the design of the study, acquisition of *in vitro* and *in vivo* data, analysis/interpretation of the data, and writing of the manuscript. PL conceived of the concept and contributed to the design of the study, analysis/interpretation of data, writing of the manuscript, and supervision of the project. JN designed and synthesized related molecules. FZ contributed to the acquisition of *in vitro* data, analysis/interpretation of the data, and writing of the manuscript. YGL and BW contributed to the design of the study. KP contributed to the analysis/interpretation of the data. All authors contributed to manuscript revision, and approved the submitted version.

## Funding

This work was supported by funding associated with an endowed chair to PL and grants from Endocyte Inc. and Umoja Biopharma Inc.

## Conflict of Interest

The authors declare that the research was conducted in the absence of any commercial or financial relationships that could be construed as a potential conflict of interest.

The authors declare that this study received funding from Endocyte Inc. and Umoja Biopharma Inc. The funder was not involved in the study design, collection, analysis, interpretation of data, the writing of this article or the decision to submit it for publication.

## Publisher’s Note

All claims expressed in this article are solely those of the authors and do not necessarily represent those of their affiliated organizations, or those of the publisher, the editors and the reviewers. Any product that may be evaluated in this article, or claim that may be made by its manufacturer, is not guaranteed or endorsed by the publisher.

## Abbreviations

TAM, tumor associated macrophage; MDSC, myeloid-derived suppressor cell; TLR7a, Toll like receptor 7 agonist; TME, tumor microenvironment; PBS, phosphate buffered saline; GFP, green fluorescent protein; FRβ, folate receptor beta.

## References

[1] Barrett DM Singh N Porter DL Grupp SA June CH. Chimeric Antigen Receptor Therapy for Cancer. Annu Rev Med (2014) 65(1):333–47. doi: 10.1146/annurev-med-060512-150254 PMC4120077 24274181

[2] Li J Li W Huang K Zhang Y Kupfer G Zhao Q. Chimeric Antigen Receptor T Cell (CAR-T) Immunotherapy for Solid Tumors: Lessons Learned and Strategies for Moving Forward. J Hematol Oncol (2018) 11(1):22–2. doi: 10.1186/s13045-018-0568-6 PMC5809840 29433552

[3] Zhang Z Liu S Zhang B Qiao L Zhang Y. T Cell Dysfunction and Exhaustion in Cancer. Front Cell Dev Biol (2020) 8:17. doi: 10.3389/fcell.2020.00017 32117960 PMC7027373

[4] Braga TT Agudelo JSH Camara NOS. Macrophages During the Fibrotic Process: M2 as Friend and Foe. Front Immunol (2015) 6:602. doi: 10.3389/fimmu.2015.00602 26635814 PMC4658431

[5] Nagaraj S Schrum AG Cho H-I Celis E Gabrilovich DI. Mechanism of T Cell Tolerance Induced by Myeloid-Derived Suppressor Cells. J Immunol (2010) 184(6):3106–16. doi: 10.4049/jimmunol.0902661 PMC2832724 20142361

[6] Massi D Marconi C Franchi A Bianchini F Paglierani M Ketabchi S. Arginine Metabolism in Tumor-Associated Macrophages in Cutaneous Malignant Melanoma: Evidence From Human and Experimental Tumors. Hum Pathol (2007) 38(10):1516–25. doi: 10.1016/j.humpath.2007.02.018 17640716

[7] Shi T Ma Y Yu L Jiang J Shen S Hou Y. Cancer Immunotherapy: A Focus on the Regulation of Immune Checkpoints. Int J Mol Sci (2018) 19(5):1389. doi: 10.3390/ijms19051389 PMC5983802 29735917

[8] Afik R Zigmond E Vugman M Klepfish M Shimshoni E Pasmanik-Chor M. Tumor Macrophages Are Pivotal Constructors of Tumor Collagenous Matrix. J Exp Med (2016) 213(11):2315–31. doi: 10.1084/jem.20151193 PMC5068227 27697834

[9] Ochando JC Chen SH. Myeloid-Derived Suppressor Cells in Transplantation and Cancer. Immunol Res (2012) 54(1-3):275–85. doi: 10.1007/s12026-012-8335-1 PMC4000733 22535241

[10] Murray PJ. Macrophage Polarization. Annu Rev Physiol (2017) 79(1):541–66. doi: 10.1146/annurev-physiol-022516-034339 27813830

[11] Ryzhov S Novitskiy SV Goldstein AE Biktasova A Blackburn MR Biaggioni I. Adenosinergic Regulation of the Expansion and Immunosuppressive Activity of CD11b+Gr1+ Cells. J Immunol (2011) 187(11):6120–9. doi: 10.4049/jimmunol.1101225 PMC3221925 22039302

[12] Limagne E Euvrard R Thibaudin M Rébé C Derangère V Chevriaux A. Accumulation of MDSC and Th17 Cells in Patients With Metastatic Colorectal Cancer Predicts the Efficacy of a FOLFOX-Bevacizumab Drug Treatment Regimen. Cancer Res (2016) 76(18):5241–52. doi: 10.1158/0008-5472.CAN-15-3164 27496709

[13] Mantovani A Allavena P Sica A Balkwill F. Cancer-Related Inflammation. Nature (2008) 454(7203):436–44. doi: 10.1038/nature07205 18650914

[14] Zhang Q-w Liu L Gong C-y Shi H-s Zeng Y-h Wang X-z. Prognostic Significance of Tumor-Associated Macrophages in Solid Tumor: A Meta-Analysis of the Literature. PLoS One (2012) 7(12):e50946–6. doi: 10.1371/journal.pone.0050946 PMC3532403 23284651

[15] Wang P-F Song S-Y Wang T-J Ji W-J Li S-W Liu N. Prognostic Role of Pretreatment Circulating MDSCs in Patients With Solid Malignancies: A Meta-Analysis of 40 Studies. Oncoimmunology (2018) 7(10):e1494113–e1494113. doi: 10.1080/2162402X.2018.1494113 30288362 PMC6169582

[16] van Dalen FJ van Stevendaal MHME Fennemann FL Verdoes M Ilina O. Molecular Repolarisation of Tumour-Associated Macrophages. Molecules (2018) 24(1):9. doi: 10.3390/molecules24010009 PMC6337345 30577495

[17] Cresswell GM Wang B Kischuk EM Broman MM Alfar RA Vickman RE. Folate Receptor Beta Designates Immunosuppressive Tumor-Associated Myeloid Cells That Can Be Reprogrammed With Folate-Targeted Drugs. Cancer Res (2021) 81(3):671–84. doi: 10.1158/0008-5472.CAN-20-1414 PMC10987201 33203700

[18] Savage P Horton V Moore J Owens M Witt P Gore ME. A Phase I Clinical Trial of Imiquimod, an Oral Interferon Inducer, Administered Daily. Br J Cancer (1996) 74(9):1482–6. doi: 10.1038/bjc.1996.569 PMC2074776 8912549

[19] Harrison LI Skinner SL Marbury TC Owens ML Kurup S McKane S. Pharmacokinetics and Safety of Imiquimod 5% Cream in the Treatment of Actinic Keratoses of the Face, Scalp, or Hands and Arms. Arch Dermatol Res (2004) 296(1):6–11. doi: 10.1007/s00403-004-0465-4 15083310

[20] Geller MA Cooley S Argenta PA Downs LS Carson LF Judson PL. Toll-Like Receptor-7 Agonist Administered Subcutaneously in a Prolonged Dosing Schedule in Heavily Pretreated Recurrent Breast, Ovarian, and Cervix Cancers. Cancer Immunol Immunother (2010) 59(12):1877–84. doi: 10.1007/s00262-010-0914-1 PMC4098785 20820775

[21] Low PS Henne WA Doorneweerd DD. Discovery and Development of Folic-Acid-Based Receptor Targeting for Imaging and Therapy of Cancer and Inflammatory Diseases. Acc Chem Res (2008) 41(1):120–9. doi: 10.1021/ar7000815 17655275

[22] Zhang F Ayaub EA Wang B Puchulu-Campanella E Li YH Hettiarachchi SU. Reprogramming of Profibrotic Macrophages for Treatment of Bleomycin-Induced Pulmonary Fibrosis. EMBO Mol Med (2020) 12(8):e12034. doi: 10.15252/emmm.202012034 32597014 PMC7411553

[23] Mendelsohn LG Gates SB Habeck LL Shackelford KA Worzalla J Shih C. The Role of Dietary Folate in Modulation of Folate Receptor Expression, Folylpolyglutamate Synthetase Activity and the Efficacy and Toxicity of Lometrexol. Adv Enzyme Regul (1996) 36:365–81. doi: 10.1016/0065-2571(96)00001-5 8869756

[24] Frigerio B Bizzoni C Jansen G Leamon CP Peters GJ Low PS. Folate Receptors and Transporters: Biological Role and Diagnostic/Therapeutic Targets in Cancer and Other Diseases. J Exp Clin Cancer Res (2019) 38(1):125–5. doi: 10.1186/s13046-019-1123-1 PMC6417013 30867007

[25] Kochenderfer JN Yu Z Frasheri D Restifo NP Rosenberg SA. Adoptive Transfer of Syngeneic T Cells Transduced With a Chimeric Antigen Receptor That Recognizes Murine CD19 can Eradicate Lymphoma and Normal B Cells. Blood (2010) 116(19):3875–86. doi: 10.1182/blood-2010-01-265041 PMC2981541 20631379

[26] Zhang B Napoleon JV Liu X Luo Q Srinivasarao M Low PS. Sensitive Manipulation of CAR T Cell Activity Using a Chimeric Endocytosing Receptor. J Immunother Cancer (2020) 8(2):e000756. doi: 10.1136/jitc-2020-000756 33127654 PMC7604868

[27] Antony AC Tang Y-S Khan RA Biju MP Xiao X Li Q-J. Translational Upregulation of Folate Receptors Is Mediated by Homocysteine *via* RNA-Heterogeneous Nuclear Ribonucleoprotein E1 Interactions. J Clin Invest (2004) 113(2):285–301. doi: 10.1172/JCI200411548 14722620 PMC310746

[28] Xia W Hilgenbrink AR Matteson EL Lockwood MB Cheng J-X Low PS. A Functional Folate Receptor Is Induced During Macrophage Activation and can be Used to Target Drugs to Activated Macrophages. Blood (2009) 113(2):438–46. doi: 10.1182/blood-2008-04-150789 18952896

[29] Basal E Eghbali-Fatourechi GZ Kalli KR Hartmann LC Goodman KM Goode EL. Functional Folate Receptor Alpha Is Elevated in the Blood of Ovarian Cancer Patients. PLoS One (2009) 4(7):e6292. doi: 10.1371/journal.pone.0006292 19617914 PMC2707611

[30] Laboratories CR. Syngeneic Models Immunotherapy Response Data. Available at: https://www.criver.com/resources/syngeneic-model-data.

[31] Zheng X Mansouri S Krager A Grimminger F Seeger W Pullamsetti SS. Metabolism in Tumour-Associated Macrophages: a. Eur Respir Rev (2020) 29(157):200134. doi: 10.1183/16000617.0134-2020 33004525 PMC9488699

[32] Marvel D Gabrilovich DI. Myeloid-Derived Suppressor Cells in the Tumor Microenvironment: Expect the Unexpected. J Clin Invest (2015) 125(9):3356–64. doi: 10.1172/JCI80005 PMC4588239 26168215

[33] Hanada T Nakagawa M Emoto A Nomura T Nasu N Nomura Y. Prognostic Value of Tumor-Associated Macrophage Count in Human Bladder Cancer. Int J Urol (2000) 7(7):263–9. doi: 10.1046/j.1442-2042.2000.00190.x 10910229

[34] De Cicco P Ercolano G Ianaro A. The New Era of Cancer Immunotherapy: Targeting Myeloid-Derived Suppressor Cells to Overcome Immune Evasion. Front Immunol (2020) 11:1680. doi: 10.3389/fimmu.2020.01680 32849585 PMC7406792

[35] Wing A Fajardo CA Posey AD Shaw C Da T Young RM. Improving CART-Cell Therapy of Solid Tumors With Oncolytic Virus-Driven Production of a Bispecific T-Cell Engager. Cancer Immunol Res (2018) 6(5):605–16. doi: 10.1158/2326-6066.CIR-17-0314 PMC6688490 29588319

[36] Guedan S Alemany R. CAR-T Cells and Oncolytic Viruses: Joining Forces to Overcome the Solid Tumor Challenge. Front Immunol (2018) 9:2460. doi: 10.3389/fimmu.2018.02460 30405639 PMC6207052

[37] Rodriguez-Garcia A Lynn RC Poussin M Eiva MA Shaw LC O'Connor RS. CAR-T Cell-Mediated Depletion of Immunosuppressive Tumor-Associated Macrophages Promotes Endogenous Antitumor Immunity and Augments Adoptive Immunotherapy. Nat Commun (2021) 12(1):877–7. doi: 10.1038/s41467-021-20893-2 PMC7873057 33563975

[38] Qian BZ Pollard JW. Macrophage Diversity Enhances Tumor Progression and Metastasis. Cell (2010) 141(1):39–51. doi: 10.1016/j.cell.2010.03.014 20371344 PMC4994190

[39] Lin Y Xu J Lan H. Tumor-Associated Macrophages in Tumor Metastasis: Biological Roles and Clinical Therapeutic Applications. J Hematol Oncol (2019) 12(1):76. doi: 10.1186/s13045-019-0760-3 31300030 PMC6626377

[40] Loeuillard E Yang J Buckarma E Wang J Liu Y Conboy C. Targeting Tumor-Associated Macrophages and Granulocytic Myeloid-Derived Suppressor Cells Augments PD-1 Blockade in Cholangiocarcinoma. J Clin Invest (2020) 130(10):5380–96. doi: 10.1172/JCI137110 PMC7524481 32663198

[41] Ugel S De Sanctis F Mandruzzato S Bronte V. Tumor-Induced Myeloid Deviation: When Myeloid-Derived Suppressor Cells Meet Tumor-Associated Macrophages. J Clin Invest (2015) 125(9):3365–76. doi: 10.1172/JCI80006 PMC4588310 26325033

[42] Oh M-H Sun I-H Zhao L Leone RD Sun I-M Xu W. Targeting Glutamine Metabolism Enhances Tumor-Specific Immunity by Modulating Suppressive Myeloid Cells. J Clin Invest (2020) 130(7):3865–84. doi: 10.1172/JCI131859 PMC7324212 32324593

[43] Korbecki J Olbromski M Dzięgiel P. Ccl18 in the Progression of Cancer. Int J Mol Sci (2020) 21(21):1–24. doi: 10.3390/ijms21217955 PMC7663205 33114763

[44] Birn H Spiegelstein O Christensen EI Finnell RH. Renal Tubular Reabsorption of Folate Mediated by Folate Binding Protein 1. J Am Soc Nephrol (2005) 16(3):608–15. doi: 10.1681/ASN.2004080711 15703271

[45] Grapp M Wrede A Schweizer M Hüwel S Galla H-J Snaidero N. Choroid Plexus Transcytosis and Exosome Shuttling Deliver Folate Into Brain Parenchyma. Nat Commun (2013) 4(1):2123–3. doi: 10.1038/ncomms3123 23828504

[46] Elnakat H Ratnam M. Distribution, Functionality and Gene Regulation of Folate Receptor Isoforms: Implications in Targeted Therapy. Adv Drug Deliv Rev (2004) 56(8):1067–84. doi: 10.1016/j.addr.2004.01.001 15094207

[47] Shen J Putt KS Visscher DW Murphy L Cohen C Singhal S. Assessment of Folate Receptor-β Expression in Human Neoplastic Tissues. Oncotarget (2015) 6(16):14700–9. doi: 10.18632/oncotarget.3739 PMC4546498 25909292

[48] Chmielewski M Abken H. CAR T Cells Releasing IL-18 Convert to T-Bet. Cell Rep (2017) 21(11):3205–19. doi: 10.1016/j.celrep.2017.11.063 29241547

[49] Eerola AK Soini Y Pääkkö P. A High Number of Tumor-Infiltrating Lymphocytes Are Associated With a Small Tumor Size, Low Tumor Stage, and a Favorable Prognosis in Operated Small Cell Lung Carcinoma. Clin Cancer Res (2000) 6(5):1875–81. 10815910

[50] Wang Z Yang Q Tan Y Tang Y Ye J Yuan B. Cancer-Associated Fibroblasts Suppress Cancer Development: The Other Side of the Coin. Front Cell Dev Biol (2021) 9:613534. doi: 10.3389/fcell.2021.613534 33614646 PMC7890026

