# Supplementary material for: Repolarization of Tumor-Infiltrating Myeloid Cells for Augmentation of CAR T Cell Therapies
Source: Front Immunol. 2022 Feb 16;13:816761. doi: 10.3389/fimmu.2022.816761 (PMC8889096; doi:10.3389/fimmu.2022.816761)
Supplement: Supplementary file 1 [file DataSheet_1.pdf]

Supplemental Figures

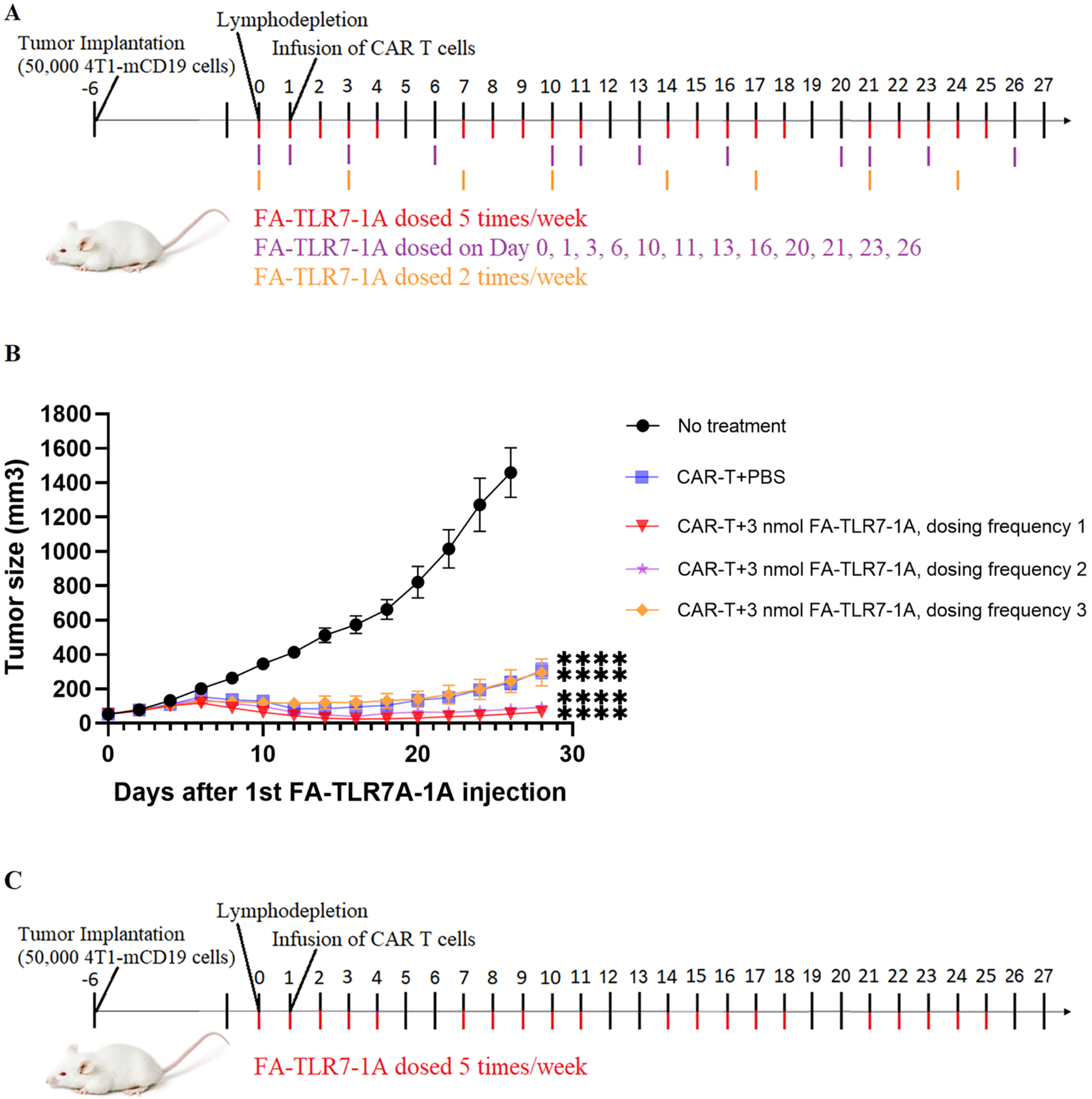

Figure S1.
Evaluation of the effect of FA-TLR7-1A dosing frequency on CAR T cell efficacy. (**A**) Timeline of tumor implantation, lymphodepletion, CAR T cell implantation, and treatments. (**B**) Analysis of the effect of different FA-TLR7-1A dosing frequencies (defined in the figure) on tumor growth. Mean ± SEM. Mean ± SEM. Statistical significance between multiple groups was determined using a 1-way ANOVA followed by a Tukey post hoc analysis. (*P < 0.05, **P < 0.01, ***P < 0.001, ****P < 0.0001). (**C**) Timeline of tumor implantation, lymphodepletion, CAR T cell implantation, and treatments for all future studies after FA-TLR7-1A dose optimization.

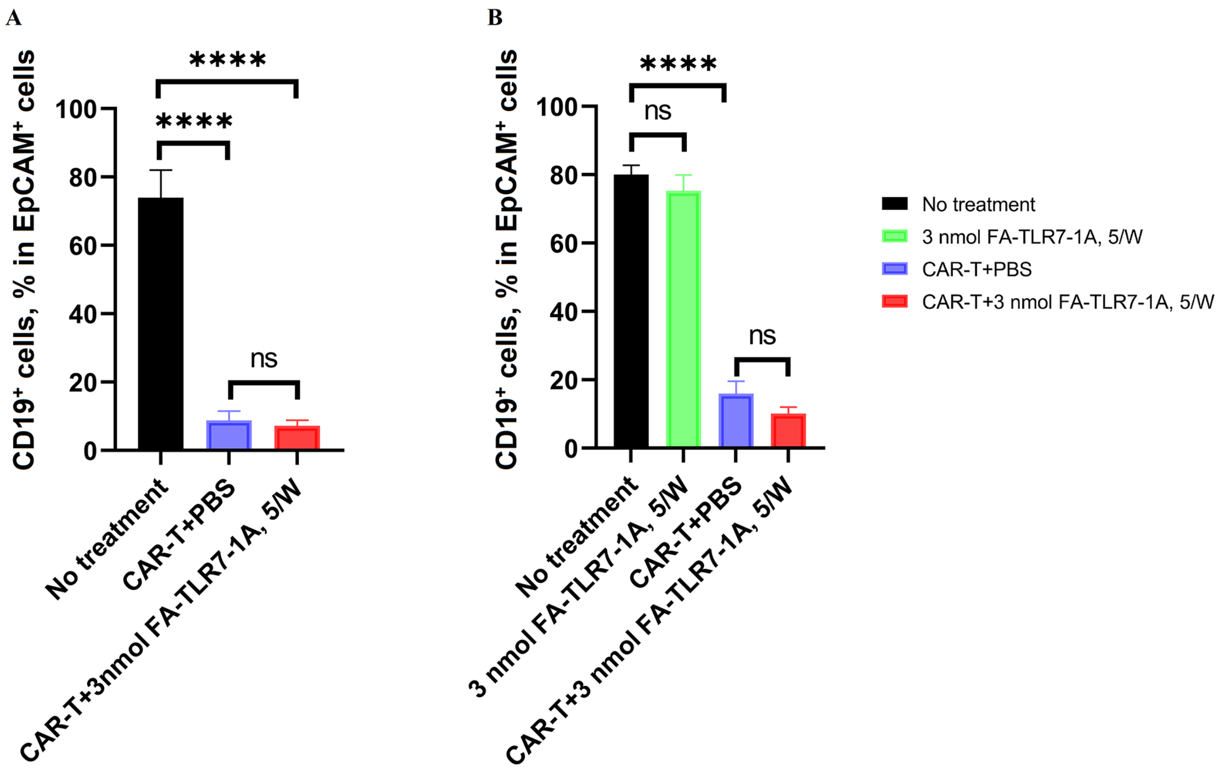

Figure S2.

Loss of CD19 expression on 4T1-mCD19 cancer cells following CAR T cell treatment of solid tumors. (A) The percent of total EpCAM positive tumor cells that were mCD19 positive at the end of the therapy shown in Fig. 3A. (**B**) The percent of total EpCAM positive tumor cells that were mCD19 positive at the end of the therapy shown in Fig. 4. The tumors in Fig. 3A were grown from a heterogeneous mixture of CD19 positive 4T1 cells whereas the tumors in Fig. 4 were grown from a single cell clone of CD19 positive 4T1 cells. Mean ± SEM. Statistical significance between multiple groups was determined using a 1-way ANOVA followed by a Tukey post hoc analysis. (*P < 0.05, **P < 0.01, ***P < 0.001, ****P < 0.0001).

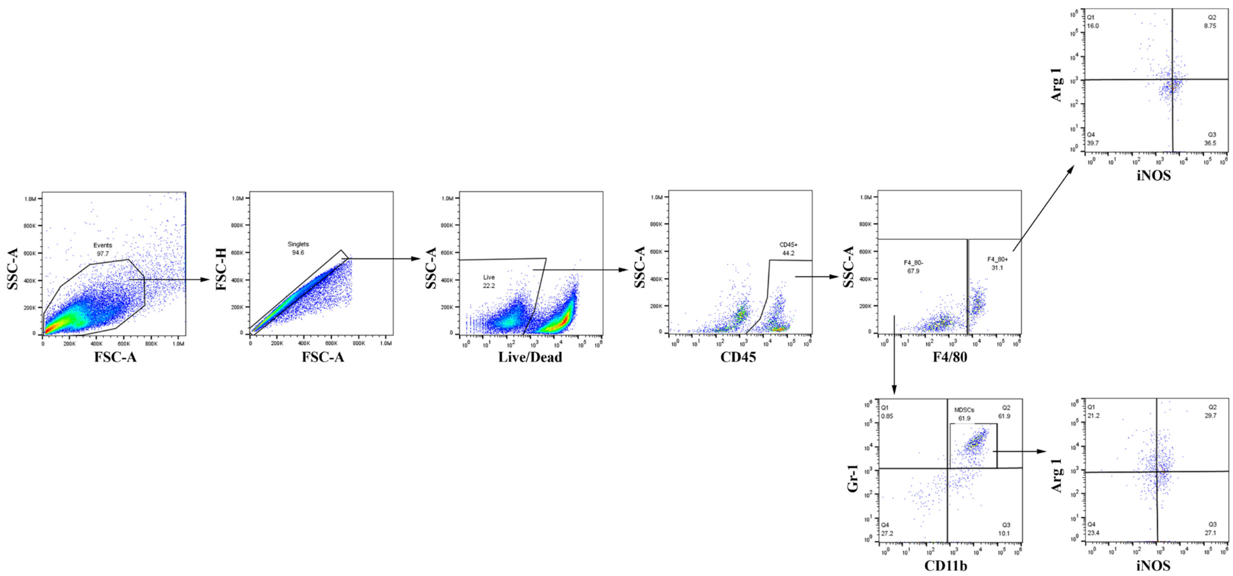

Figure S3.

Flow cytometry gating strategy. Representative flow cytometry plots from tumor digested cells showing the gating scheme for macrophages and MDSCs. Total tumor cell singlets were first gated by forward versus side scatter (FSC-A)/SSC-A), after which aggregates were excluded by FSC-A vs. FSC-H. Next, live cells were selected with Zombie Violet and immune cells were gated with CD45 positive staining. These were then further gated for the subsets of interest, namely, F4/80^{+} cells (macrophages) and F4/80^{-} CD11b^{+} Gr1^{+} (MDSCs). Macrophages were further divided into iNOS^{+} Arg 1^{-} − M1 macrophages, and iNOS^{-} Arg 1^{+} − M2 macrophages.

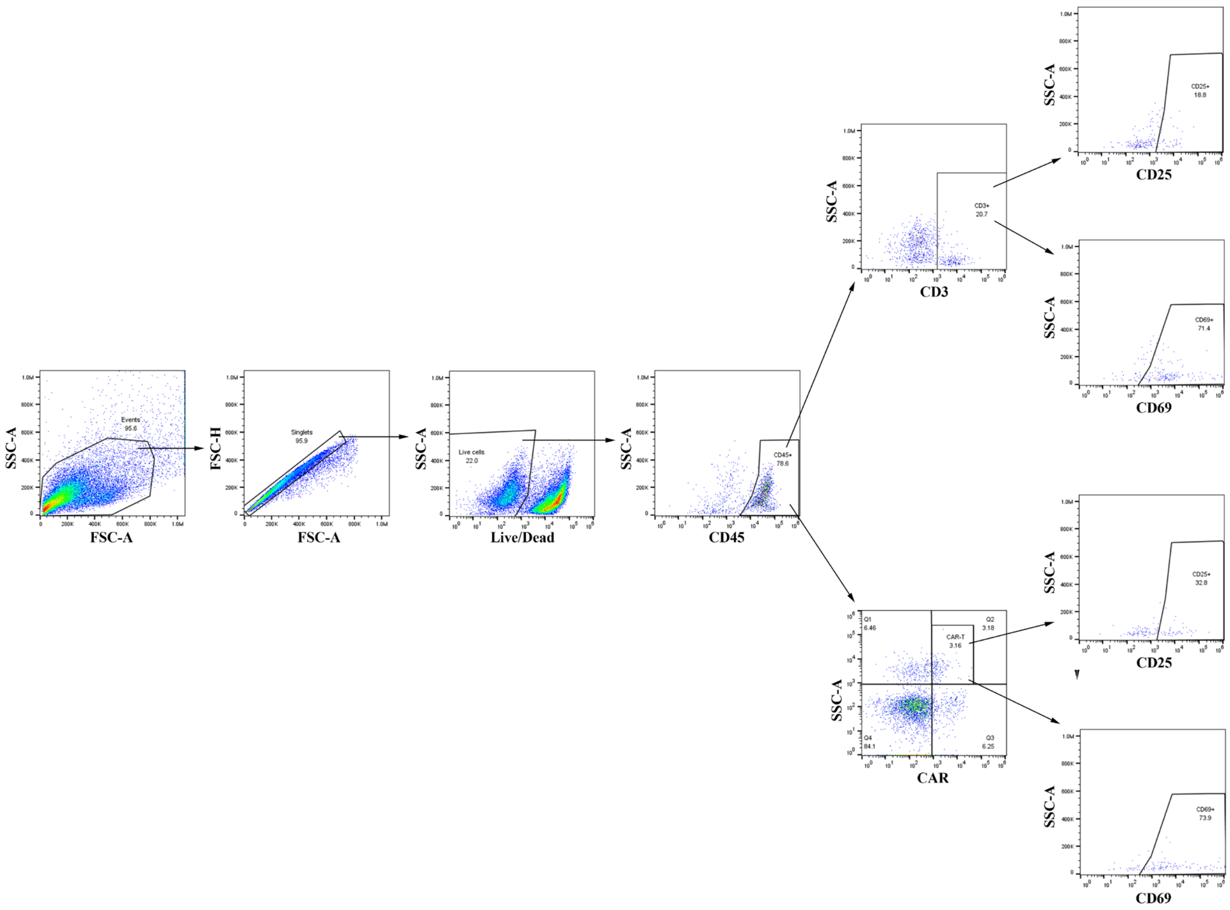

Figure S4.

Flow cytometry gating strategy. Representative flow cytometry plots from tumor digested cells showing the gating scheme for total T cells and CAR T cells. Analysis was performed as in Fig. S3 except CD45 positive cells were further gated for the subsets of interest, namely, $CD3^+$ – total T cells, and $CD3^+$ CAR^+ – CAR T cells. Each subset was further gated on $CD25^+$ or $CD69^+$ for the activated sub-populations.

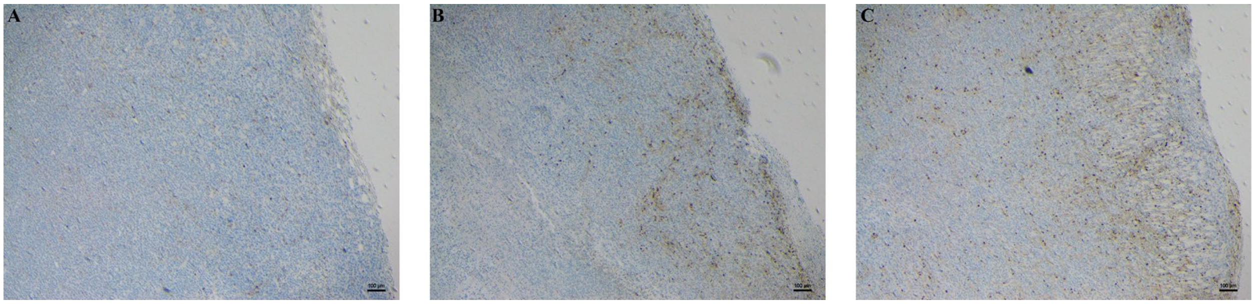

Figure S5.

IHC staining of mouse CD3 in 4T1-mCD19 tumors (4x). (A) No treatment; **(B)** CAR-T+PBS**; (C)**. CAR-T + 3nmol FA-TLR7-1A, 5/W.

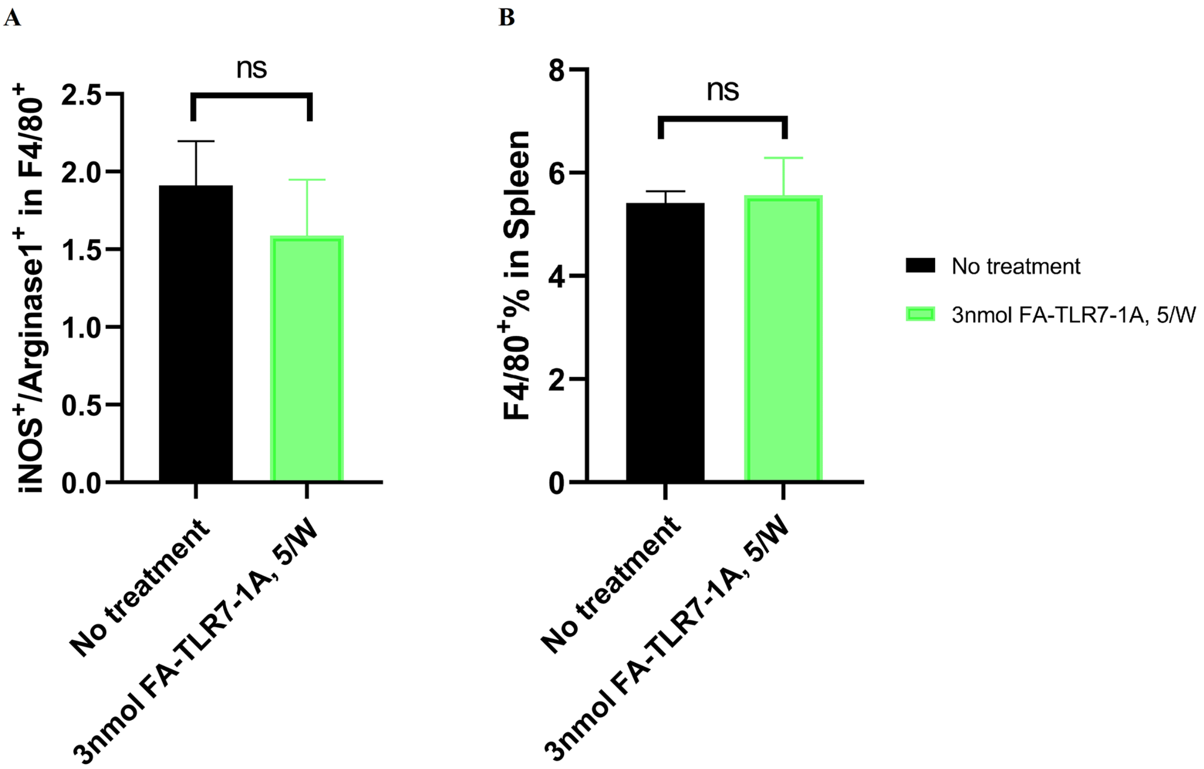

Figure S6.

Examination of the effect of FA-TLR7-1A on the splenic macrophages from the mice treated in Fig. 4.
The M1:M2 ratio and the percentage of macrophages in the spleen after FA-TLR7-1A treatment were analyzed by flow cytometry as described in Fig. 5. Mean ± SEM. Statistical significance between groups was determined using an unpaired two-tailed t-test (*P < 0.05).
